# Editorial: Diabetes and Obesity Effects on Lung Function

**DOI:** 10.3389/fendo.2020.00462

**Published:** 2020-07-16

**Authors:** Xiao-Feng Chen, Liang-Jun Yan, Albert Lecube, Xiaoqiang Tang

**Affiliations:** ^1^School of Basic Medical Sciences, Chengdu University of Traditional Chinese Medicine, Chengdu, China; ^2^Department of Pharmaceutical Sciences, University of North Texas System College of Pharmacy, University of North Texas Health Science Center, Fort Worth, TX, United States; ^3^Obesity, Diabetes and Nutrition Research Group, Endocrinology and Nutrition Department, Institut de Recerca Biomèdica de Lleida (IRBLleida), University Hospital Arnau de Vilanova de Lleida, Universitat de Lleida, Lleida, Spain; ^4^Key Laboratory of Birth Defects and Related Diseases of Women and Children of MOE, State Key Laboratory of Biotherapy, West China Second University Hospital, Sichuan University, Chengdu, China

**Keywords:** diabetes, insulin, pulmonary disease, lung function, molecular mechanisms

Diabetes mellitus is a group of metabolic diseases characterized by persistent hyperglycemia which prevalence and incidence have risen sharply worldwide (1, 2). In particular, over 90% of all diabetes cases are type 2 diabetes mellitus (T2DM) (3, 4). Nowadays, studies have shown that absolute or relative insufficient insulin secretion and impaired action of insulin signaling pathways are two of the major causes of diabetes. Moreover, diabetes is a disease affecting a lot of organs such as kidney, liver, eyes, and heart and there is also a growing body of evidence suggesting that the lung is also one of the target organs in diabetes (5, 6). This special issue Research Topic, “Diabetes and Obesity Effects on Lung Function” contains a collection of studies that are mainly focused on understanding the association between diabetes and pulmonary diseases, and the underlying mechanisms. We hope that the paper collected in this special issue would broaden our knowledge about diabetic lung and facilitate the development of better disease management strategies.

Circadian rhythms are 24-h rhythms in physiology driven by the circadian clock system of the suprachiasmatic nucleus located in the hypothalamus (7). Circadian rhythms are widely involved in metabolism-related diseases (8, 9). As Sirtuins are adenine dinucleotide (NAD^+^)-dependent histone deacetylases (10), it has been suggested that Sirtuins also play significant roles in regulating diseases such as type 2 diabetes (11, 12). Zhou et al. provided a detailed review discussing the role of circadian rhythms and Sirtuins in the diabetic lung. After an initial description of the histological and functional changes that appears in the diabetic lung, they describe the circadian clock that affects insulin secretion and insulin sensitivity and protects the diabetic lung against oxidative stress and inflammation processes. Furthermore, they illustrated the potential regulatory mechanism of Sirtuins (SIRT1, SIRT3, and SIRT6) in the lung of subjects with diabetes together with the interactions between Sirtuins and the circadian clock. This work improves our understanding of the circadian regulation of metabolic pulmonary diseases.

Wang et al. discussed the potential mechanisms involved in idiopathic pulmonary fibrosis (IPF) contributed by diabetes and provided an overview of diabetic IPF. Specifically, they concluded that diabetes is a risk factor and a marker of poor prognosis for IPF patients through discussing epidemiology, pulmonary functional changes, and pathological changes. Mechanistically, persistent hyperglycemia could destroy the alveolar epithelial cells or increase the production of pro-inflammatory and pro-fibrotic factors.

Lung cancer remains the most common cancer (11.6% of all) and the leading cause of cancer deaths, with over 1.7 million deaths worldwide in 2018 (13). Chin-Hsiao Tseng contributes with a retrospective cohort study examining the influence of insulin on lung cancer in patients with type 2 diabetes. Surprisingly, the study demonstrates that insulin use would accelerate the progression of lung cancer. This study significantly improves the understanding of lung cancer and introduces a warning signal regarding insulin therapy in these patients.

Accumulating evidence has shown that disturbed sleep is implicated in the physiopathology of type 2 diabetes (14, 15). And sympathetic hyperactivity is associated with type 2 diabetes as well as sleep apnea-hypopnea syndrome (SAHS). López-Cano et al. concluded that intermittent hypoxia resulting from sleep breathing disorders increases the sympathetic activity in patients with type 2 diabetes, which in turn amplifies the effect of hyperglycemia, increasing both cardiovascular risk and microangiopathic diabetic complications. Thus, a good sleep could help prevent and treat diabetes and cardiovascular diseases.

Lung inflammation is also controlled by metabolic regulators. The review paper submitted by Xu et al. focuses on understanding the effect of oxidative stress and the AMPK-Nrf2 pathway in pneumonia. Specifically, this study discusses how ROS accumulation mediated by a pulmonary lesion or metabolic diseases such as type 2 diabetes or obesity makes the lung more susceptible to bacterial and viral infection. In particular, activation AMPK-Nrf2 could ameliorate pneumonia through its antioxidative effects.

In summary, papers collected in this special issue underline the deleterious roles of diabetes mellitus and metabolic regulators in pulmonary diseases including idiopathic pulmonary fibrosis, lung cancer, and pneumonia. It stresses that more attention should be paid to illustrate the underlying mechanisms that may provide invaluable insights into novel approaches for attenuating diabetic lung injury in the future.

## Author Contributions

All authors listed have made a substantial, direct and intellectual contribution to the work, and approved it for publication.

## Conflict of Interest

The authors declare that the research was conducted in the absence of any commercial or financial relationships that could be construed as a potential conflict of interest.
